# Staphylococcal PknB as the First Prokaryotic Representative of the Proline-Directed Kinases

**DOI:** 10.1371/journal.pone.0009057

**Published:** 2010-02-04

**Authors:** Malgorzata Miller, Stefanie Donat, Sonja Rakette, Thilo Stehle, Thijs R. H. M. Kouwen, Sander H. Diks, Annette Dreisbach, Ewoud Reilman, Katrin Gronau, Dörte Becher, Maikel P. Peppelenbosch, Jan Maarten van Dijl, Knut Ohlsen

**Affiliations:** 1 Department of Medical Microbiology, University Medical Center Groningen and University of Groningen, Groningen, The Netherlands; 2 Institut für Molekulare Infektionsbiologie, Universität Würzburg, Würzburg, Germany; 3 Department of Cell Biology, University Medical Center Groningen and University of Groningen, Groningen, The Netherlands; 4 Interfakultäres Institut für Biochemie, Universität Tübingen, Tübingen, Germany; 5 Institut für Mikrobiologie, Ernst-Moritz-Arndt Universität Greifswald, Greifswald, Germany; 6 Department of Pediatrics, Vanderbilt University School of Medicine, Nashville, Tennessee, United States of America; National Institute of Allergy and Infectious Diseases, National Institutes of Health, United States of America

## Abstract

In eukaryotic cell types, virtually all cellular processes are under control of proline-directed kinases and especially MAP kinases. Serine/threonine kinases in general were originally considered as a eukaryote-specific enzyme family. However, recent studies have revealed that orthologues of eukaryotic serine/threonine kinases exist in bacteria. Moreover, various pathogenic species, such as *Yersinia* and *Mycobacterium*, require serine/threonine kinases for successful invasion of human host cells. The substrates targeted by bacterial serine/threonine kinases have remained largely unknown. Here we report that the serine/threonine kinase PknB from the important pathogen *Staphylococcus aureus* is released into the external milieu, which opens up the possibility that PknB does not only phosphorylate bacterial proteins but also proteins of the human host. To identify possible human targets of purified PknB, we studied *in vitro* phosphorylation of peptide microarrays and detected 68 possible human targets for phosphorylation. These results show that PknB is a proline-directed kinase with MAP kinase-like enzymatic activity. As the potential cellular targets for PknB are involved in apoptosis, immune responses, transport, and metabolism, PknB secretion may help the bacterium to evade intracellular killing and facilitate its growth. In apparent agreement with this notion, phosphorylation of the host-cell response coordinating transcription factor ATF-2 by PknB was confirmed by mass spectrometry. Taken together, our results identify PknB as the first prokaryotic representative of the proline-directed kinase/MAP kinase family of enzymes.

## Introduction

Despite their clinical relevance, the mechanisms employed by pathogenic bacteria to subvert the host immune system remain only partially characterised. It has become clear, however, that pathogens create a beneficial environment for their survival by secreting proteins that mimic the functions of several host proteins. One of the best known bacterial examples is *Yersinia pestis*, the plague bacterium, which injects its effector proteins into the host cells by the type III secretion system [1], [2]. These *Yersinia* effector (Yop) proteins include the eukaryotic-like serine/threonine kinase YpkA, also known as YopO [3]. This kinase shows a high degree of sequence similarity to mammalian serine/threonine protein kinase domains. YpkA is translocated into a host cell where it disrupts the actin-based cytoskeletal system and promotes both survival and replication of bacteria by an unknown mechanism [3], [4], [5]. Nevertheless, the full spectrum of human proteins that are phosphorylated by YpkA has remained elusive so far [6].

Eukaryotic-like serine/threonine protein kinases (STPKs) are present not only in the *Yersinia* genus, but they have also been identified in the soil microorganism *Myxococcus xanthus* [7], [8] and in human pathogens, such as *Mycobacterium tuberculosis*, which even encodes 11 STPKs. Only two of these (PknG and PknK) are soluble proteins, while the other nine STPKs contain a transmembrane domain [9]. Moreover STPKs have also been identified in *Pseudomonas aeruginosa* [10], [11], *Streptococcus pneumoniae* [12], [13], [14] and in *Staphylococcus aureus* [15], [16], [17]. However, the precise biological functions and substrate specificities of these kinases have not yet been defined.

Recently, attention has been focussed on the PknB kinase of *S. aureus*. This Gram-positive bacterium is part of the human microbiota, but it can turn into a dangerous pathogen, causing a wide range of infections [18], [19], [20]. Although *S. aureus* is mostly considered as an extracellular pathogen, it can invade a variety of mammalian non-professional cells, such as nasal endothelial cells. Moreover *S. aureus* survives phagocytosis by professional phagocytes [21], [22], [23], such as neutrophils [24], [25], mouse or rat macrophages [26], [27], [28], [29], and human macrophages [30], [31]. To overcome the stressful conditions imposed by its host, *S. aureus* has evolved various protective and offensive responses [32], [33], [34], [35], such as sensing of environmental stimuli and the activation and inactivation of response regulators [36], [37]. This is generally achieved through cascades of phosphorylation reactions in the host, which focuses a strong interest on the role of kinases, such as the serine/threonine kinase PknB (also known as StpK) in staphylococcal persistence.

The PknB kinase is composed of three extracellular PASTA domains (penicillin binding domains), a central transmembrane domain and an intracellular kinase domain [16], [38]. Interestingly, it was recently reported that PknB is not only involved in regulation of the central metabolism of *S. aureus* [15], but also determines staphylococcal infection of mouse kidneys in an abscess model [17]. The latter observation raises the question whether the kinase activity of PknB is directly or indirectly involved in the pathogenicity of *S. aureus*. A direct role of PknB in infection is conceivable since serine/threonine kinases play key roles in mammalian cell signalling, and at least two bacterial equivalents, YpkA of *Yersinia* and PknG of *Mycobacterium tuberculosis*, have been shown to be directly involved in the subversion of host cells during the respective infectious processes [39], [40]. However the exact role played by PknB in pathogenesis or staphylococcal persistence has thus far remained unclear.

Here we show that full-size soluble PknB is present in the medium of growing *S. aureus* cells. We therefore investigated whether PknB of *S. aureus* can recognize and phosphorylate known substrates of human serine/threonine kinases. For this purpose, we used peptide microarrays with known human phosphorylation sites. The phosphorylation profile and mass spectrometry results show that PknB is a proline-directed kinase, which can indeed phosphorylate specific human targets. The observed target specificity of PknB indicates possible roles for this enzyme in a wide range of host cell signalling processes during *S. aureus* infection.

## Results and Discussion

### Identification of Extracellular PknB

**Figure 1:**
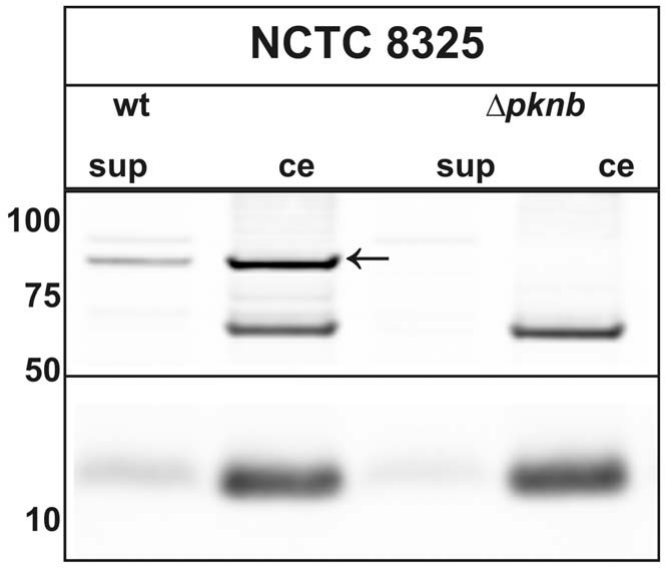
Release of PknB into the growth medium of *S. aureus*. The *S. aureus* strain NCTC 8325 (wt) or a Δ*pknB* derivative were propagated at 37°C in TSB and harvested at OD_600_ 2. Crude extracts (ce) and supernatant (sup) fractions were isolated, corrected for OD and separated by NuPAGE electrophoresis (Invitrogen). Two-fold higher amounts of the supernatant fractions were used for PAGE as compared to the crude extracts. Immunoblotting was conducted using specific antibodies against PknB (upper panel) or TrxA (lower panel). The latter served as an indicator for cell lysis. The position of the specific PknB signal is marked with a black arrow. The band at ∼60 kDA corresponds to an unidentified protein, which cross-reacts with the antibodies against PknB. The molecular weight of marker proteins is indicated on the left.

It has previously been reported that different bacteria such as *M. tuberculosis* and *Yersinia* species can secrete their eukaryotic-like serine/threonine kinases directly into the host. This mechanism allows these bacteria to survive intracellularly [9], [39], to disrupt the actin cytoskeleton [41], or to cause host cell apoptosis [40]. Since these bacterial ser/thr kinases need to be exported in order to impact on host cells, we investigated whether PknB might be detectable in the extracellular milieu of *S. aureus*. As shown by Western blotting using polyclonal antibodies against PknB, the full-size PknB was detectable both in the cellular and growth medium fractions of cells of *S. aureus* NCTC 8325 harvested at an OD_600_ of 2. As expected, PknB was neither detectable in cellular nor growth medium fractions of the Δ*pknB* mutant. The precise mechanism by which PknB is liberated from the wild-type cells remains to be elucidated. However, there is precedence for the release of membrane proteins, or fragments thereof, into the extracellular milieu of Gram-positive bacteria, such as *S. aureus*, by as yet unknown mechanisms [42]. One possibility is that these proteins are released by cell lysis, remaining stable in the medium due to an intrinsic resistance to extracytoplasmic proteolysis [43]. The idea that PknB is released by lysis would be consistent with the detection of relatively small amounts of the cytoplasmic marker protein thioredoxin A (TrxA) in the growth medium fractions (Fig. 1). TrxA is a cytoplasmic bacterial protein, which acts as an antioxidant by facilitating the reduction of cysteine disulfides in other cytoplasmic proteins. Since TrxA is normally a cytoplasmic protein, it will only be found in the extracellular milieu when bacterial cells have lysed. Notably, compared to the cellular samples, we detected relatively more extracellular PknB than extracellular TrxA, which might suggest that the release of PknB is the consequence of a specific process rather than cell lysis.

Irrespective of the mechanism by which PknB is released into the extracellular milieu of *S. aureus*, its release may impact on human host cell functions. This could be the case upon internalization of *S. aureus*. Although *S. aureus* is primarily an extracellular pathogen, there is strong evidence that it can be internalized by a wide range of human host cells. For example, *S. aureus* invades non-professional phagocytes by a mechanism which requires a specific interaction between the bacterial fibronectin-binding protein and the host cell [44], [45], [46]. This leads to host signal transduction, activation of tyrosine kinases, cytoskeletal rearrangement and endosome uptake. Bayles and Qazi reported that internalized *S. aureus* is able to escape from the host endosome, and this fact opens up the possibility of direct interactions of released PknB with proteins of the human host [47], [48]

### Eukaryotic Phosphorylation Sites Recognized by PknB

Peptide arrays (PepChips) have previously been used successfully to profile the activity of kinases in eukaryotic cell lysates [49]. We therefore employed this array-based technology to investigate whether the staphylococcal kinase PknB has the ability to recognize and phosphorylate human phosphorylation sites. When the PepChips were incubated with purified and active PknB [16] and [^33^P-γ] ATP, radioactivity was efficiently incorporated in a particular subset of the peptides on the chip. In contrast, little radioactivity was incorporated when the arrays were incubated with [^33^P-γ] ATP in the absence of PknB. We identified 68 potential substrates for PknB, of which the biological functions are summarised in Table 1 (for details, see Table S1). Interestingly, 32 of the potential human substrates of PknB are involved in signal transduction and cell communication. The identified peptides include serine/threonine kinases, cell cycle control proteins, and regulators of protein phosphorylation such as adaptor molecules. Our results suggest that any active PknB released from invasive *S. aureus* cells may target signal transduction mechanisms for host cell subversion. In addition, 13 potential PknB substrates are involved in gene regulation, including transcription factors, transcription regulatory proteins and RNA binding proteins. Three potential substrates play a role in immune responses and recognition, five in transport processes, ten in cell growth and maintenance (cytoskeletal and structural proteins), two in cell metabolism, two in stress responses, and one in apoptosis.

**Table 1:** Cellular processes that can be targeted by PknB.

**Transport** **Total: 5 proteins**	**Cell growth and maintanance** **Total: 10 proteins**
membrane transport proteins, voltage gated channel, water channel proteins	structural proteins, cell cycle regulation proteins, cell adhesion proteins
**Metabolism** **Total: 2 proteins**	**Immune response and recognition** **Total: 3 proteins**
phosphotransferase, ribosomal subunit	immunoglobulin, cell surface receptors
**Signal transduction and Cell communication** **Total: 32 proteins**	**Regulation of nucleobase, nucleoside, nucleotide and nucleic acid metabolism** **Total: 13 proteins**
receptors, cell cycle proteins, cell junction proteins, serine/threonine kinases, tyrosine kinases, transport/cargo proteins, adapter molecules	DNA binding site, RNA binding site, ribonucleoproteins, transcription factors, transcription regulatory proteins
**Apoptosis** **Total: 1 protein**	**Stress** **Total: 2 proteins**
Bcl2- interacting protein BIM	Heat-shock proteins

Functional classes of human proteins that are potentially phosphorylated by PknB, as identified in the present PepChip analysis, are classified by their biological function.

Phosphorylation of the identified potential human PknB targets would result in significant changes in host cell signal transduction. One of the peptides that is best phosphorylated by PknB (^64^IVADQTPTPTR^74^) is derived from the Activating Transcription Factor-2 (ATF-2) (see Table S2). ATF-2 belongs to the bZIP (Basic Leucine Zipper Domain) DNA-binding protein family and is expressed by almost all human cells (GeneNote, Ensemlb ID: ENSG00000115966). In unstimulated cells, ATF-2 is maintained in an inactive form by interactions between its own activation domain and its bZIP domain [50]. In response to certain stimuli [51], [52], [53], the kinases p38 and JNK phosphorylate ATF-2 at amino acids Thr69 and Thr71 [52], [53]. The phosphorylated ATF-2 can then form homodimers and heterodimers [54], which bind with high affinity to the consensus sequence 5′-TGACGTCA-3′ [55] in target gene promoters, resulting in their activation. The phosphorylation of ATF-2 thus results in the expression of a broad spectrum of proteins implicated in different processes, such as cell cycle molecules (cyclin D1) [56], cell adhesion molecules [57], growth factors [58], anti-apoptotic factors [59], and invasion-related molecules [60].

**Figure 2:**
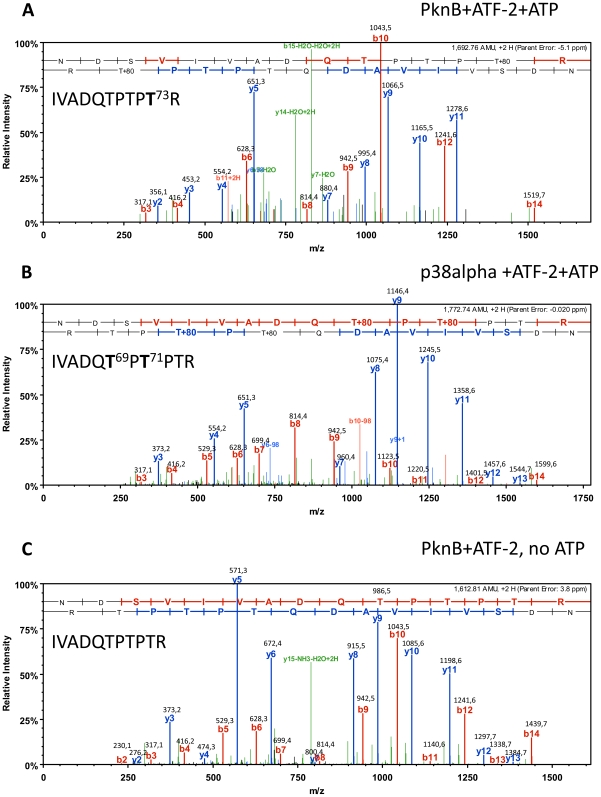
Verification of PknB-dependent phosphorylation of ATF-2. Recombinant ATF-2 was incubated with PknB (A) or p38 (B) in kinase reaction buffer for 30 minutes at 37°C. As a control, ATF-2 was incubated with PknB in the absence of ATP. After tryptic digestion, the resulting peptides were analyzed via online-mass spectrometry. The panels show the spectra for the ATF-2 peptide VIVADQTPTPTR that was either phosphorylated at the Thr73 by PknB, or Thr 69 and Thr 71 by p38. The b- and y-ions are high-lighted and the observed masses are given. Also the peptide sequence is indicated and amino acids that have been identified my mass spectrometric analysis are indicated in bold letters. The upper sequence corresponds to the b- and the lower sequence to the y-ions.

To confirm that PknB is able to phosphorylate ATF-2, we incubated ATF-2 with PknB *in vitro* and performed mass spectrometric analyses. As a positive control we incubated ATF-2 with p38. The results show that PknB can indeed phosphorylate ATF-2 (Fig. 2). However, PknB phosphorylated Thr73, whereas p38 phosphorylated Thr69 and Thr71. Interestingly, Thr73 is the known phosphorylation site of the Human Vaccinia-related Kinase 1 (VRK1) [61]. VRK1 is a ser/thr kinase overexpressed in proliferating cells [62], [63]. Phosphorylation of ATF-2 on Thr 73 by VRK1 kinase leads to the activation of ATF-2 and, consequently, the induction of cellular protection mechanisms.

Another peptide that was very efficiently phosphorylated by PknB belongs to the Bcl-2 interacting protein Bim (see Table S2). *In vivo* this peptide is recognized by the c-Jun NH_2_-terminal kinase (JNK) (Table S1) [64], [65]. Bim is a member of the pro-apoptotic Bcl-2 family of proteins, which play a critical role in apoptosis regulation. A short peptide motif, DKSTQT^56^P, which is present in BimL and BimEL, but absent from BimS, mediates the binding of Bim to the LC8 cytoplasmic dynein light chain. Importantly, the same motif is also recognized and phosphorylated by PknB (Table S1). Exposure of cells to stress causes the activation of JNK kinase, which phosphorylates Bim at Thr56 in the afore-mentioned DKSTQTP motif. The phosphorylation leads to conformational changes in Bim and subsequent dissociation of Bim from dynein motor complexes [66]. The activated Bim may directly activate pro-apoptotic Bax, or indirectly activate Bax by binding anti-apoptotic Bcl-2 family proteins (e.g., Bcl-2 and Bcl-X_L_) [67], [68]. Judged by the observed phosporylation of the DKSTQT^56^P peptide, the release of PknB from invasive *S. aureus* cells might have similar effects as its phosphorylation by JNK.

A third intriguing target of PknB identified through phosphorylation profiling is the cytoskeleton-associated protein paxillin. This protein has previously been reported as a target for the phosphatase YopH, which is injected by *Yersinia* into human host cells, thereby affecting cytoskeleton integrity [69]. Taken together, the identified potential targets of PknB are fully consistent with previously reported effects of *S. aureus* and other bacteria on host cell apoptosis [70], [71], [72], [73] and a wide range of cellular processes [4], [6], [13], [39], [40], [74], [75].

Interestingly, a comparison of the identified human phosphorylation sites of PknB with staphylococcal proteins revealed about 300 putative PknB target sequences in proteins of *S. aureus* (data not shown). These include amino acid sequences in proteins that are known to be phosphorylated by PknB, such as triosephosphate isomerase, DnaK, elongation factors, ribosomal proteins and trigger factor [15].

### PknB Is a Proline-Directed Serine/Threonine Kinase

**Figure 3:**
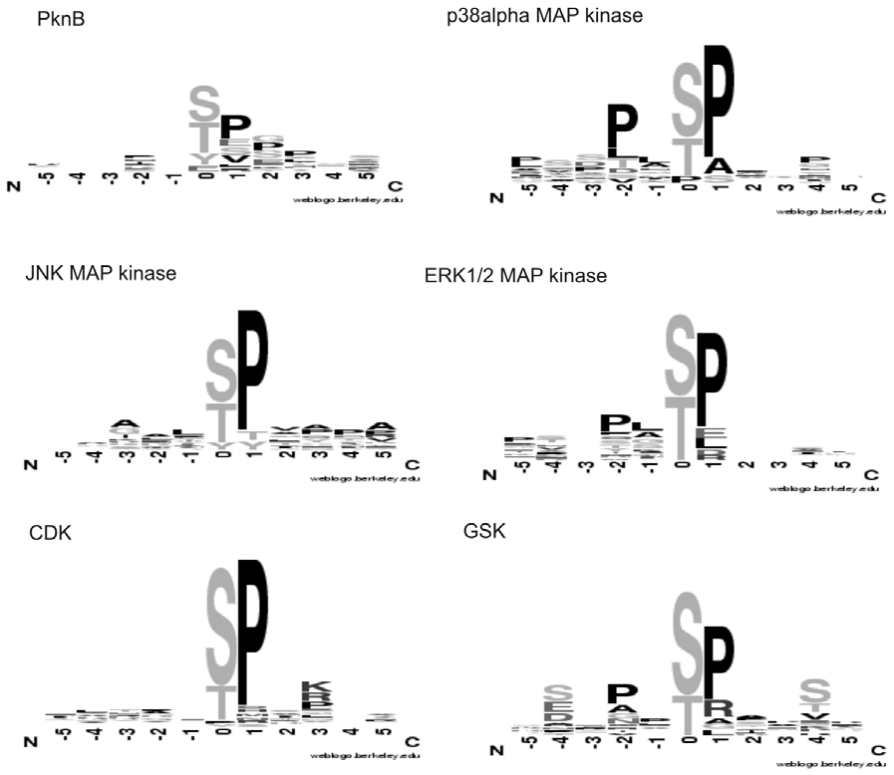
Sequence logo of PknB phosphorylation sites and comparison to known phosphorylation sites of human kinases. The image shows consensus recognition sites for the staphylococcal PknB and other proline-directed ser/thr kinases.

To determine which amino acids are preferably phosphorylated by PknB, we generated a sequence logo based on the 15% best-phosphorylated peptides (Fig. 3). The most frequently found amino acids in PknB-phosphorylated peptides are serine and threonine. This observation is in agreement with the serine/threonine kinase signature in the primary sequence of PknB. Apparently, the signature has portability across the eukaryotic/prokaryotic divides. Nevertheless tyrosine phosphorylation can also be unambiguously identified making this study the first demonstration of enzymatic tyrosine kinase activity in a single isolated prokaryotic enzyme. What is also clearly evident in the sequence logo is the presence of a proline residue next to phosphorylated serine, threonine or tyrosine residues. Thus, it seems that proline is part of the PknB recognition and target sequence. This links PknB to the evolutionary well-conserved family of proline-directed kinases, which includes cyclin-dependent protein kinases (CDKs), mitogen-activated protein kinases (MAP kinases) and glycogen synthase kinase-3 (GSK-3; Fig. 3). These kinases play a crucial role in cell cycle, transcription, signal transduction and are involved in many diseases like cancer or Alzheimer's disease [76], [77], [78]. Like the eukaryotic MAP kinases, PknB has the ability to phosphorylate ATF-2 at least *in vitro*, which implies that PknB has a MAP kinase-like enzymatic activity. This view is supported by the observation that PknB is also involved in the regulation of important cellular functions in *S. aureus*, including central metabolic pathways [15] and cell wall metabolism [79]. Taken together, our results imply that PknB is the first prokaryotic representative of the proline-directed kinases, a cardinal family of regulators of eukaryotic cellular physiology. A major challenge for future studies will be to identify human proteins that are phosphorylated by PknB *in vivo*, for example upon internalization of *S. aureus* by macrophages.

## Materials and Methods

### Detection of Extracellular PknB by Immunoblotting

*S. aureus* NCTC 8325 or a *pknB* mutant of this strain [16] were cultivated in 10 ml of TSB (37°C, 250 rpm) and growth was monitored by OD_600_ readings. Samples of 3 ml were harvested at OD_600_ 2 and cells were separated from the growth medium by centrifugation (8000 rpm, 5 min, 4°C). Bacterial cells were washed in PBS, resuspended in sample buffer (NuPage, Invitrogen) and disrupted using a Precellys 24 bead beater (three times 30 s, 6800 rpm; Bertin Technologies). Proteins from the growth medium fraction were collected by TCA-acetone precipitation [80]. Protein samples were mixed with gel-loading buffer with reducing agent and incubated for 5 min at 95°C. To receive a clear signal for the supernatant fractions twice as much as from the crude extract was applied to gel electrophoresis. The proteins were separated on a 10% Bis-Tris gel (Invitrogen) at 200 V for 35 min in NuPAGE® MES SDS Running Buffer (Invitrogen). The separated proteins were transferred to a nitrocellulose membrane (Protran®, Schleicher and Schuell) by semi-dry blotting at 200 mA for 75 min. Membranes were blocked for one hour in Blocking Buffer (Odyssey, Li-Cor biosciences). Rabbit primary antibodies against the kinase domain of PknB were added (1∶5000 in blocking buffer) and membranes were incubated for 1 hour. Next, membranes were washed 3 times for 5 minutes in PBS-T (Phosphate Buffered Saline Tween-20) before adding a fluorescent secondary antibody at a 1∶20000 dilution in blocking buffer (IRDye 800 CW goat anti-rabbit antibody from LiCor biosciences). Membranes were incubated for 1 hour in the dark, washed three times for 5 min in PBS-T and once in PBS. After transferring the membranes into fresh PBS, they were scanned using the Odyssey Infrared Imaging System (LiCor Biosciences). As a cell lysis control, antibodies against cytoplasmic protein TrxA were used in the same concentration as PknB antibodies.

### Cloning, Expression and Purification of *S. aureus* TrxA

All procedures for DNA purification, restriction, ligation, agarose gel electrophoresis, and transformation of competent *E. coli* DH5α cells were carried out as previously described [81]. Genomic DNA of *S. aureus* RN4220 [82] was isolated using the Genelute Bacterial Genomic DNA kit (Sigma). The *trxA* gene on this genomic DNA was PCR-amplified using the primers GGGGGCATATGGCAATCGTAAAAGTAA and GGGGGCTCGAGTAAATGTTTATCTAAAACTTC. The PCR product was cloned into *Hinc*II (*Hind*II) restricted pUC18 [83]. After verification of the sequence, the *trxA* gene was excised from this plasmid with *Nde*I and *Xho*I, and ligated into the same restriction sites of pET26b(+) (Novagen, Inc.), downstream of the T7 promoter and upstream of an in-frame His(6)tag sequence. The resulting pET26-SatrxA plasmid was checked by sequencing and used to transform *E. coli* BL21(DE3) (Invitrogen) for high-level *trxA* expression and purification. 10 ml of an overnight culture of this strain was used to inoculate 1 liter fresh LB medium and grown until an OD_600_ of 0.7 was reached. Then, isopropylthiogalactoside (IPTG) was added to a final concentration of 1 mM to induce TrxA production. After 3 hrs of induction, cells were harvested by centrifugation and resuspended in binding buffer (20 mM NaPi, 300 mM NaCl, 10% (v/v) glycerol, 5 mM imidazole, 3 mM DTT, pH 7.4). Next, cells were disrupted by two passages through a French Press (2500 PSI). Cellular debris was removed by centrifugation (30 min at 30000 g, 4°C), and the clarified supernatant fraction was applied to a nickel-charged IMAC column (5 ml HisTrap HP, GE Healthcare). Unbound sample was washed from the column with binding buffer using an ÄKTA explorer (GE Healthcare). Next, the His-tagged TrxA protein was eluted from the column using binding buffer with a gradient of increasing imidazole concentrations (up to 500 mM imidazole). The eluted fractions were checked for the presence of pure TrxA protein using SDS-PAGE and subsequent silver-staining. Further purification was achieved by concentrating the proteins with Vivaspin columns (Vivascience) and loading them on a Superdex 75 gel filtration column (Amersham) pre-equilibrated with 20 mM NaPi, 150 mM NaCl, 10% glycerol and 3.5 mM DTT, pH 7.4. Fractions containing the purified TrxA proteins were pooled and dialyzed 3 times against 20 mM Tris-HCl, pH 7.6, with 150 mM NaCl. Specific polyclonal antibodies against the purified TrxA protein were raised in rabbits (Eurogentec).

### Kinome Array Analysis

PepChip™ Kinomics slides containing 976 fully annotated, disease-related kinase phosphorylation sites in triplicate (Pepscan, Lelystad, The Netherlands, http://www.pepscan.com/) were incubated with 50 µl of the ser/thr kinase PknB incubation mix (0.8 µg/ml PknB kinase domain, 60 mM HEPES, pH 7.5, 3 mM MgCl_2_, 3 mM MnCl_2_, 1 mM DTT, 50% glycerol, 50 µM ATP supplemented with 1Mbq [γ-^33^P] ATP, 0.03% Brij-35, 50 µg/ml bovine serum albumin, 3 mM Na_3_VO_4_, 50 µg/ml PEG 8000) for 90 min in a humidified incubator. As a negative control we used the “empty” incubation mix without PknB. After incubation the peptide arrays were washed twice in 2 M NaCl (1% Tween-20) and PBS-T. Next, the arrays were rinsed twice in demineralised water and air-dried. The dried slides were transferred to a phosphor imager plate (Fuji Storm 860, Stanford, GE, USA) and exposed for 72 hours. The density of the spots was measured and analyzed with array software.

### Peptide Array Data Analysis

To analyze the intensity of spots and to correct for background phosphorylation, the ScanAnalyze software and grid tools were used, and the resulting data were exported to an excel sheet. Three replicate data sets were taken for further statistical analysis. To this end, the Spearman correlation coefficient was calculated for each combination of the three sets. The average and standard deviation for each peptide were determined and plotted in an amplitude-based hierarchical fashion. If only background phosphorylation is present, this amplitude-based distribution can be described by a single exponent. Thus, determining the exponent describing amplitude behavior of the 500 least phosphorylated peptides should give an adequate description of array background phosphorylation and, in practice, this was indeed the case. 125 Peptides which exhibited the incorporation of γ-^33^P in the absence of added kinase were excluded from further analysis. Peptides of which the average phosphorylation minus 1.96 times the standard deviation was higher as the value expected from describing the background distribution were considered to represent true phosphorylation events. Two-sided heteroscedastic t-tests were also performed on each set of values to determine significance (p<0.05).

### Sequence Logos

Sequence logos were created with the weblogo server at http://weblogo.berkeley.edu/logo.cgi using either the preferred substrates of PknB, or known phosphorylation sequences for human kinases as available at http://www.phosphosite.org/homeAction.do

### In Vitro Phosphorylation of ATF-2 by Staphylococcal PknB

In order to confirm the phosphorylation of ATF-2 by PknB, the *in vitro* assay was performed. Purified staphylococcal kinase PknB (26 µg) was incubated with 50 µg of Activating Transcription Factor fusion protein (Cell Signaling) in kinase incubation mix (50 mM HEPES, 1 mM DTT, 0.01 Brij35, 3 mM MnCl_2_, 3 mM MgCl_2_, 50 µM ATP) for 30 minutes at 37°C in waterbath. As a positive control, 0.1 µg of p38-alpha MAP kinase (Cell Signaling) was incubated with 50 µg of ATF-2. As negative controls, the following reaction mixtures were used: PknB with ATF-2 but without ATP; PknB with ATP but without ATF-2; and ATF-2 with ATP but without PknB.

### Phosphorylation Site Identification and Protein Identification by Mass Spectrometry

Trypsin (Promega) was activated by 15 min incubation at 30°C in activation buffer and then added 1∶200 to the samples, digestion was allowed to proceed over night at 37°C. The resulting peptides were separated by liquid chromatography and measured online by ESI-mass spectrometry using a nanoACQUITY UPLC™ system (Waters, Milford, MA) coupled to an LTQ Orbitrap™ mass spectrometer (Thermo Fisher Scientific, Waltham, MA). Peptides were desalted onto a trap column (Symmetry® C18, Waters). Elution was performed onto an analytical column (BEH130 C18, Waters) by a binary gradient of buffer A (0.1% (v/v) acetic acid) and B (100% (v/v) acetonitrile, 0.1% (v/v) acetic acid) over a period of 50 min with a flow rate of 400 nl/min. The LTQ Orbitrap was operated in data-dependent MS/MS mode using MSA for phospho-relevant masses. Proteins were identified by searching all MS/MS spectra in .dta format against all *S. aureus* NCTC 8325 proteins and added ATF-2 protein (extracted from the NCBI database) using Sorcerer™-SEQUEST® (ThermoFinnigan, San Jose, CA; version v.27, rev. 11). Sequest was searched with a fragment ion mass tolerance of 1.00 Da and a parent ion tolerance of 10 ppm. Up to two missed tryptic cleavages were allowed. Methionine oxidation (+15.99492 Da), Carbamidomethylation (+57.021465 Da) and phosphorylation of STY (+79.966331 Da) was set as variable modification. Proteins were identified by at least two peptides applying a stringent SEQUEST filter. Sequest identifications required at least deltaCn scores of greater than 0.10 and XCorr scores of greater than 1.9, 2.2, 3.75 and 3.75 for singly, doubly, triply and quadruply charged peptides. Phosphorylated peptides which passed this filter were examined manually and accepted only, when b- or y- ions confirmed the phosphorylation site.

## Supporting Information

Table S1 PknB-phosphorylated peptides
(0.15 MB DOC)

Table S2 Peptides phosphorylated by PknB grouped according to function
(0.16 MB DOC)
