# Supplementary material for: Staphylococcal PknB as the First Prokaryotic Representative of the Proline-Directed Kinases
Source: PLoS One. 2010 Feb 4;5(2):e9057. doi: 10.1371/journal.pone.0009057 (PMC2816222; doi:10.1371/journal.pone.0009057)
Supplement: Table S1 — PknB-phosphorylated peptides (0.15 MB DOC) [file pone.0009057.s001.doc]

| Scanalyze  spot# | PEPTIDE | PROTEIN | UPSTREAM KINASE | p-value | phosphorylated amino acid |
| --- | --- | --- | --- | --- | --- |
| 597 | IVADQTPTPTR | Activating transcription factor 2 | JNK2;MAPK14 | 3,4E-02 | T51 |
| 481 | PQPPKSPGPHS | RAD9 | JNK | 2,3E-02 | S336 |
| 689 | RSNPPSRKGSG | Connexin 32 | CDK | 3,3E-02 | S229 |
| 673 | QPRCTSLDSAL | MAP3K8 | AKT1 | 3,9E-02 | S400 |
| 875 | STQTPSPPCQA | BCL2-interacting protein BIM | JNK1 | 3,3E-02 | S118 |
| 981 | RKRRPTSGLHP | BRCA1 | AKT1 | 9,8E-03 | S509 |
| 669 | LVEPLTPSGEA | EGF receptor | ERK1;ERK2 | 4,0E-02 | T693 |
| 745 | SATIVSPPPSS | E2F transcription factor 1 | MAPK ,CDC2,PKA,MAPK,CaMK,RSK2 | 5,7E-03 | S332 |
| 458 | RRCKHYVELLV | Vanilloid receptor like channel 2 | Lyn | 2,6E-03 | Y253 |
| 404 | EEGEGYEEPDS | CD19 | ABL | 4,6E-04 | Y508 |
| 832 | PIGEDEESESD | Vesicular monoamine transporter 2 | CK1;CK2 | 3,3E-03 | S511 |
| 881 | GEKRASSPFRR | Nucleolar phosphoprotein p130 | PKA | 1,4E-02 | S623 |
| 663 | NNAIETVSINN | Dopamine receptor D1 | PKA | 7,5E-03 | T360 |
| 658 | DATGDTPGAED | MAPK8 interacting protein 1 | JNK1 | 8,7E-03 | T284 |
| 926 | CRIGGSRRERS | EP4 receptor | PKC | 3,6E-03 | S354 |
| 586 | RESSVYDISEH | NR2B | CaMKII | 1,6E-03 | S383 |
| 353 | RTAASSLALVS | Uridine nucleotide receptor | nd | 2,8E-03 | S334 |
| 577 | DDEDCYGNYDN | 3-Phosphoinositide dependent protein kinase 1 | c-Src,ERK | 1,9E-04 | Y373 |
| 962 | FIGEHYVHVNA | Hepatocyte growth factor receptor | HGFR | 7,8E-04 | Y1349 |
| 672 | PEPGPYAQPSV | CRK | ABL;EGFR;IGF-I_receptor | 4,8E-03 | Y221 |
| 751 | IKRLRSQVQVS | Hepatocyte nuclear factor 4-alpha | AMPK | 2,4E-03 | S304 |
| 975 | ENFDDYMKEVG | Fatty acid binding protein 4 | Tyrosyl_kinase | 2,7E-03 | Y20 |
| 729 | KLVQASEELLR | Nucleoside diphosphate kinase 3 | nd | 3,3E-03 | S61 |
| 916 | IGDELYLEPLE | RAD9 | ABL | 7,3E-03 | Y28 |
| 841 | DKQVEYLDLDL | GAB1 | Insulin_receptor;EGFR;HGFR | 1,9E-03 | Y627 |
| 679 | HGSRHSLASTD | Low density lipoprotein receptor-related protein 1 | PKA | 2,6E-03 | S4520 |
| 491 | RKAKRSLAPRF | PKR | PKR | 7,4E-05 | S242 |
| 332 | MNEVTYSTLNF | Carcinoembryonic antigen-related cell adhesion molecule 1 | MAP3K10 | 1,6E-04 | S508 |
| 677 | LKQGASPNVQD | Cyclin dependent kinase inhibitor 2D | nd | 1,4E-03 | S66 |
| 707 | EEEHVYSFPNK | Paxillin | FAK | 7,1E-04 | Y118 |
| 205 | SFLQRYSSDPT | EGF receptor | EGFR | 2,3E-03 | Y1069 |
| 329 | ETNNDYETADG | CD32 | Lyn;Blk;Fyn;SYK | 5,8E-04 | Y279 |
| 925 | SVSVETQGDDW | Hematopoietic cell specific LYN substrate 1 | CK2alpha_1 | 2,0E-04 | T16 |
| 932 | IIRQPSEEEII | PEA15 | Akt;CaMKII | 1,8E-03 | S116 |
| 626 | FRRQLSEPCNS | ETS variant gene 1 | Ribosomal protein S6 kinase; ERBB2; Ribosomal S6 kinase 1; potassium voltage gated channel subfamily A member 2; PKA | 5,7E-03 | S191 |
| 576 | VEDNRSQVETD | AQP4 | CK2 | 3,9E-03 | S285 |

**Table S1. PknB-phosphorylated peptides**

| Scanalyze spot# | MOTIF | PROTEIN | UPSTREAM KINASE | p-value | phosphorylated amino acid |
| --- | --- | --- | --- | --- | --- |
| 461 | DGENIYIRHSS | Erythrocyte membrane protein band 41 | EGFR | 2,5E-04 | Y660 |
| 218 | DPRLLSPQQPA | Myocyte specific enhancer factor 2D | ERK5 | 3,6E-04 | S180 |
| 604 | QEQESSGEEDS | Protein phosphatase inhibitor 2 | CK2 | 1,5E-05 | S121 |
| 639 | GLQMGSNRGAS | Transgelin | PKC | 7,4E-05 | S181 |
| 15 | TSSVLYTAVQP | PDGF receptor, beta | PDGFRbeta | 2,2E-03 | Y1009 |
| 54 | YSQGASPQPQH | Cut like 1 | CDC2 | 8,2E-04 | S1237 |
| 220 | TGLYKSQRPCV | BTEB2 | PKC | 1,1E-03 | S153 |
| 616 | DEDACSDTEAT | Protein phosphatase inhibitor 2 | CK2 | 3,6E-04 | S87 |
| 676 | LTRIPSAKKYK | PEA15 | PKC | 9,1E-04 | S104 |
| 923 | AWTADSGEGDF | Fibrinogen, alpha chain | nd | 1,0E-03 | S22 |
| 819 | GSHSNSFRLSN | Protein tyrosine phosphatase, receptor type, alpha | PKCdelta | 3,6E-04 | S189 |
| 994 | GQVIMSIRTKL | Ribosomal protein L10 | Ribosomal_protein_L10 | 2,0E-03 | S137 |
| 643 | KEVKRYQCTFE | Metal regulatory transcription factor 1 | Tyrosine_kinase | 7,6E-04 | Y140 |
| 272 | KEDPIYDEPEG | Docking protein 1 | Insulin_receptor | 4,8E-04 | Y362 |
| 595 | DNTPHTPTPFK | B-Myb | CDK2 | 9,2E-04 | T518 |
| 94 | RLRQGTLRRDL | Epithelial calcium channel 2 | PKCalpha | 4,7E-05 | T702 |
| 344 | RRGDSYDLKDF | VAV1 | PKA | 2,1E-03 | Y441 |
| 889 | KSKKYSDVEVP | Adducin 1 | PKA | 5,7E-06 | S408 |
| 686 | ENTFPSPKAIP | Nude like protein | CDK5/p35 | 4,5E-03 | S231 |
| 1010 | RSYVSSGEMMV | Glial fibrillary acidic protein | PKC; RHO kinase; CAMKII | 2,4E-03 | S17 |
| 98 | SDEVQSPVRVR | PCTAIRE protein kinase 1 | CDK 5 | 1,2E-03 | S95 |
| 800 | RPESFTTPEGP | HBP | nd | 8,4E-05 | T61 |
| 407 | PEETQTQDQPM | HSP 90A | dsDNA-activated protein kinase | 2,5E-05 | T7 |
| 619 | GLMQQQKSFR | SHP2 | PKCalpha;PKCbeta1;PKCbeta2;PKCeta | 1,5E-03 | S591 |
| 66 | PSPKSYENLWF | Colony-stimulating factor 3 receptor | Hck | 3,7E-04 | Y814 |
| 177 | NQNSSSDSEAE | T-cell transcription factor 4 | CK2 | 1,2E-06 | S60 |
| 893 | QHRSSSSAPHH | Calcium channel voltage dependent beta 2 subunit | DNA-dependent protein kinase catalytic subunit | 1,3E-03 | S478 |
| 865 | LRSEFSPSVDA | c-Mos | nd | 1,0E-03 | S16 |
| 965 | SSTHYYLLPER | ACK | nd | 3,2E-04 | Y858 |
| 533 | RRRRPTPAMLF | Protein phosphatase 1, regulatory subunit 1B | PKA | 1,2E-04 | T34 |
| 499 | RDKEVSDDEAE | HSP 90A | CK2alpha 1 | 2,2E-06 | S231 |
