# Supplementary material for: Staphylococcal PknB as the First Prokaryotic Representative of the Proline-Directed Kinases
Source: PLoS One. 2010 Feb 4;5(2):e9057. doi: 10.1371/journal.pone.0009057 (PMC2816222; doi:10.1371/journal.pone.0009057)
Supplement: Table S2 — Peptides phosphorylated by PknB grouped according to function (0.16 MB DOC) [file pone.0009057.s002.doc]

|  | | PEPTIDE | PROTEIN | UPSTREAM KINASE | MOLECULAR PROCESESS |
| --- | --- | --- | --- | --- | --- |
| **Apoptosis** | 1 | STQTPSPPCQA | BCL2-interacting protein BIM | JNK1 | Adapter molecule,Receptor signaling complex scaffold activity |
| **Cell growth/maintenance** | 2 | KSKKYSDVEVP | Adducin 1 | PKA | Structural protein |
|  | 3 | DGENIYIRHSS | Erythrocyte membrane protein band 41 | EGFR | Structural protein |
|  | 4 | AWTADSGEGDF | Fibrinogen, alpha chain | nd | Cell Adhesion, Structural Protein |
|  | 5 | RSYVSSGEMMV | Glial fibrillary acidic protein | PKC;RHO_kinase;CAMKII | Structural protein |
|  | 6 | EEEHVYSFPNK | Paxillin | FAK | Cytoskeletal associated protein,Cytoskeletal protein binding |
|  | 7 | QEQESSGEEDS | Protein phosphatase inhibitor 2 | CK2 | Regulation of cell cycle |
|  | 8 | NSKRDYTGCST | PZR | SHP2 | Regulating cell migration |
|  | 9 | PQPPKSPGPHS | RAD9 | nd | Regulation of cell cycle |
|  | 10 | IGDELYLEPLE | RAD9 | ABL (ATM) | Regulation of cell cycle |
|  | 11 | GLQMGSNRGAS | Transgelin | PKC | Structural protein |
| **Immune response** | 12 | MNEVTYSTLNF | Carcinoembryonic antigen-related cell adhesion molecule 1 | MAP3K10 | Immunoglobulin,Antigen binding |
|  | 13 | EEGEGYEEPDS | CD19 | ABL | Cell surface receptor, Receptor activity |
|  | 14 | ETNNDYETADG | CD32 | Lyn;Blk;Fyn;SYK | Cell surface receptor |
| **Metabolism** | 15 | KLVQASEELLR | Nucleoside diphosphate kinase 3 | nd | Phosphotransferase |
|  | 16 | GQVIMSIRTKL | Ribosomal protein L10 | Ribosomal protein L10 | Ribosomal subunit |
| **Regulation of nucleobase, nucleoside, nucleotide and nucleic acid metabolism** | 17 | IVADQTPTPTR | Activating transcription factor 2 | JNK2;MAPK14 | Transcription factor |
|  | 18 | DNTPHTPTPFK | B-Myb | CDK2 | Transcription factor activity |
|  | 19 | RKRRPTSGLHP | BRCA1 | AKT1 | Transcription regulatory protein |
|  | 20 | TGLYKSQRPCV | BTEB2 | PKC | Transcription factor activity |
|  | 21 | YSQGASPQPQH | Cut like 1 | CDC2 | Transcription factor activity |
|  | 22 | SATIVSPPPSS | E2F transcription factor 1 | CDC2 | Transcription factor activity |
|  | 23 | FRRQLSEPCNS | ETS variant gene 1 | Ribosomal protein S6 kinase alpha5; ERBB2; Ribosomal S6kinase1; Potassium voltage gated channel subfamily A member 2; PKA | Transcription factor activity |
|  | 24 | RPESFTTPEGP | HBP | nd | RNA binding protein |
|  | 25 | DATGDTPGAED | MAPK8 interacting protein 1 | JNK1 | Transcription factor activity |
|  | 26 | KEVKRYQCTFE | Metal regulatory transcription factor 1 | Tyrosine kinase | Transcription factor activity |
|  | 27 | DPRLLSPQQPA | Myocyte specific enhancer factor 2D | ERK5 | Transcription factor activity |
|  | 28 | GEKRASSPFRR | Nucleolar phosphoprotein p130 | PKA | Transcription factor activity |
|  | 29 | NQNSSSDSEAE | T-cell transcription factor 4 | CK2 | Transcription factor activity |

**Table S2. Peptides phosphorylated by PknB grouped according to function**

|  | | MOTIF | PROTEIN | UP-KINASE | MOLECULAR PROCESESS |
| --- | --- | --- | --- | --- | --- |
| **Signal transduction,Cell communication** | 30 | DDEDCYGNYDN | 3-Phosphoinositide dependent protein kinase 1 | c-Src | Serine/threonine kinase |
|  | 31 | SSTHYYLLPER | ACK | nd | tyrosine kinase |
|  | 32 | LRSEFSPSVDA | c-Mos | nd | Protein threonine/tyrosine kinase activity |
|  | 33 | PSPKSYENLWF | Colony-stimulating factor 3 receptor | Hck | Cytokine receptor, Transmembrane receptor activity |
|  | 34 | PEPGPYAQPSV | CRK | ABL; EGFR; IGF-I receptor | Adapter molecule,Receptor signaling complex scaffold activity |
|  | 35 | LKQGASPNVQD | Cyclin dependent kinase inhibitor 2D | nd | Cell cycle control protein,Regulation of cell cycle |
|  | 36 | KEDPIYDEPEG | Docking protein 1 | Insulin receptor | Adapter molecule,Receptor signaling complex scaffold activity |
|  | 37 | NNAIETVSINN | Dopamine receptor D1 | Nd | G protein coupled receptor |
|  | 38 | LVEPLTPSGEA | EGF receptor | ERK1; ERK2 | Transmembrane receptor protein tyrosine kinase activity |
|  | 39 | SFLQRYSSDPT | EGF receptor | EGFR | Transmembrane receptor protein tyrosine kinase activity |
|  | 40 | CRIGGSRRERS | EP4 receptor | PKC | G protein coupled receptor |
|  | 41 | ENFDDYMKEVG | Fatty acid binding protein 4 | Tyrosyl_kinase | Chaperone |
|  | 42 | DKQVEYLDLDL | GAB1 | Insulin receptor; EGFR; HGFR | Adapter molecule,Receptor signaling complex scaffold activity |
|  | 43 | SVSVETQGDDW | Hematopoietic cell specific LYN substrate 1 | CK2alpha 1 | Unclassified |
|  | 44 | FIGEHYVHVNA | Hepatocyte growth factor receptor | HGFR | Transmembrane receptor protein tyrosine kinase activity |
|  | 45 | IKRLRSQVQVS | Hepatocyte nuclear factor 4-alpha | AMPK | Nuclear receptor,Ligand-dependent nuclear receptor activity |
|  | 46 | HGSRHSLASTD | Low density lipoprotein receptor-related protein 1 | PKA | Cell surface receptor |
|  | 47 | QPRCTSLDSAL | MAP3K8 | AKT1 | Protein threonine/tyrosine kinase activity,Dual specificity kinase |
|  | 48 | RESSVYDISEH | NR2B | CaMKII | Extracellular ligand gated channel |
|  | 49 | ENTFPSPKAIP | Nude like protein | CDK5/p35 | Cell cycle control protein,Regulation of cell cycle |
|  | 50 | SDEVQSPVRVR | PCTAIRE protein kinase 1 | CDK 5 | Serine/threonine kinase |
|  | 51 | TSSVLYTAVQP | PDGF receptor, beta | PDGFR beta | Transmembrane receptor protein tyrosine kinase activity |
|  | 52 | IIRQPSEEEII | PEA15 | Akt; CaMKII | Transport/cargo protein |
|  | 53 | LTRIPSAKKYK | PEA15 | PKC | Transport/cargo protein |
|  | 54 | RKAKRSLAPRF | PKR (RNA-dependent protein kinase) | PKR | Protein kinase |

|  | | MOTIF | PROTEIN | UP-KINASE | MOLECULAR PROCESESS |
| --- | --- | --- | --- | --- | --- |
| **Signal transduction**  **Cell communication** | 55 | RRRRPTPAMLF | Protein phosphatase 1, regulatory subunit 1B | PKA | Regulatory/other subunit |
|  | 56 | DEDACSDTEAT | Protein phosphatase inhibitor 2 | CK2 | Cell cycle control protein,Regulation of cell cycle |
|  | 57 | GSHSNSFRLSN | Protein tyrosine phosphatase, receptor type, alpha | PKC delta | Receptor signaling protein tyrosine phosphatase activity |
|  | 58 | GLMQQQKSFR | SHP2 | PKCalpha; PKCbeta1; PKCbeta2; PKCeta | Tyrosine phosphatase |
|  | 59 | RTAASSLALVS | Uridine nucleotide receptor | Nd | G protein coupled receptor |
|  | 60 | RRCKHYVELLV | Vanilloid receptor like channel 2 | Lyn | involved in the transmission and modulation of pain, as well as the integration of diverse painful stimuli. |
|  | 61 | RRGDSYDLKDF | VAV1 | PKA | Guanine nucleotide exchange factor |
| **Transport** | 62 | VEDNRSQVETD | AQP4 aquaporin | CK2 | Water channel |
|  | 63 | QHRSSSSAPHH | Calcium channel voltage dependent beta 2 subunit | DNA-dependent protein kinase catalytic subunit | Voltage gated channel |
|  | 64 | RSNPPSRKGSG | Connexin 32 | PKC; PKA | Membrane transport protein |
|  | 65 | RLRQGTLRRDL | Epithelial calcium channel 2 | PKC alpha |  |
|  | 66 | PIGEDEESESD | Vesicular monoamine transporter 2 | CK1; CK2 | Membrane transport protein,Auxiliary transport protein |
| **Stress** | 67 | PEETQTQDQPM | HSP 90A | dsDNA-activated protein kinase |  |
|  | 68 | RDKEVSDDEAE | HSP 90A | CK2 alpha 1 |  |
